# Cardiorenal Syndrome in the Elderly: Challenges and Considerations

**DOI:** 10.3390/geriatrics10040104

**Published:** 2025-08-04

**Authors:** Matthew Jarocki, Sophie Green, Henry H. L. Wu, Rajkumar Chinnadurai

**Affiliations:** 1 Department of Renal Medicine, Northern Care Alliance NHS Foundation Trust, Salford M6 8HD, UK; matthew.jarocki2@nca.nhs.uk (M.J.); sophie.green@nca.nhs.uk (S.G.); 2 Renal Research Laboratory, Kolling Institute of Medical Research, Royal North Shore Hospital, The University of Sydney, Sydney, NSW 2065, Australia; honlinhenry.wu@health.nsw.gov.au; 3 Faculty of Biology, Medicine and Health, The University of Manchester, Manchester M1 7HR, UK

**Keywords:** cardiorenal syndrome, elderly, chronic kidney disease, heart failure

## Abstract

Cardiorenal syndrome (CRS) is a term used to describe the combined dysfunction of the heart and kidneys. This complex disorder is widely acknowledged to be challenging in both its diagnosis and management, and this is the case particularly in the elderly population, due to multi-morbidity, polypharmacy, and age-related physiological changes. Given advancements in medicine and more prolonged cumulative exposure to risk factors in the elderly population, it is likely that the prevalence of chronic kidney disease (CKD) and heart failure (HF) will continue to rise going forward. Hence, understanding the mechanisms involved in the development of CRS is paramount. There are five different CRS types—they are categorised depending on the primary organ involved the acuity of disease. The pathophysiological process behind CRS is complex, involving the interplay of many processes including hemodynamic changes, neurohormonal activation, inflammation, oxidative stress, and endothelial dysfunction and vascular stiffness. The numerous diagnostic and management challenges associated with CRS are significantly further exacerbated in an elderly population. Biomarkers used to aid the diagnosis of CRS, such as serum creatinine and brain natriuretic peptide (BNP), can be challenging to interpret in the elderly population due to age-related renal senescence and multiple comorbidities. Polypharmacy can contribute to the development of CRS and therefore, before initiating treatment, coordinating a patient-centred, multi-speciality, holistic review to assess potential risks versus benefits of prescribed treatments is crucial. The overall prognosis of CRS in the elderly remains poor. Treatments are primarily directed at addressing the sequelae of the underlying aetiology, which often involves the removal of fluid through diuretics or ultrafiltration. Careful considerations when managing elderly patients with CRS is essential due to the high prevalence of frailty and functional decline. As such, in these patients, early discussions around advance care planning should be prioritised.

## 1. Introduction

Cardiorenal syndrome (CRS) is a term associated with the combined dysfunction of the heart and kidneys. Historically, CRS has been defined according to which organ is triggering the dysfunction in the other. However, as the pathogenic pathways are so closely intertwined, this is often difficult to distinguish in clinical practice. With an ageing global population and increased cumulative exposure to risk factors associated with chronic kidney disease (CKD) and heart failure (HF), it is likely that the prevalence of CKD and HF will continue to rise. This complex disorder is widely acknowledged to be challenging to both diagnose and manage, and this is particularly so in the elderly population due to multi-morbidity, polypharmacy and age-related physiological changes.

### 1.1. Pathophysiology of Cardiorenal Syndrome

CRS is categorised into five distinct types, depending on the primary organ involved and the acute or chronic nature of the condition:

Type 1: A rapid decline in cardiac function resulting in a decrease in renal function (“Acute cardiorenal syndrome”).
Type 2: Chronic cardiac dysfunction resulting in a sustained reduction in renal function (“Chronic cardiorenal syndrome”).
Type 3: A rapid decline in renal function resulting in an acute reduction in cardiac function (“Acute renocardiac syndrome”).
Type 4: Chronic decline in kidney function resulting in chronic cardiac dysfunction (“Chronic renocardiac syndrome”).
Type 5: Systemic diseases resulting in both cardiac and renal dysfunction (“Secondary cardiorenal syndrome”).

**Figure 1:**
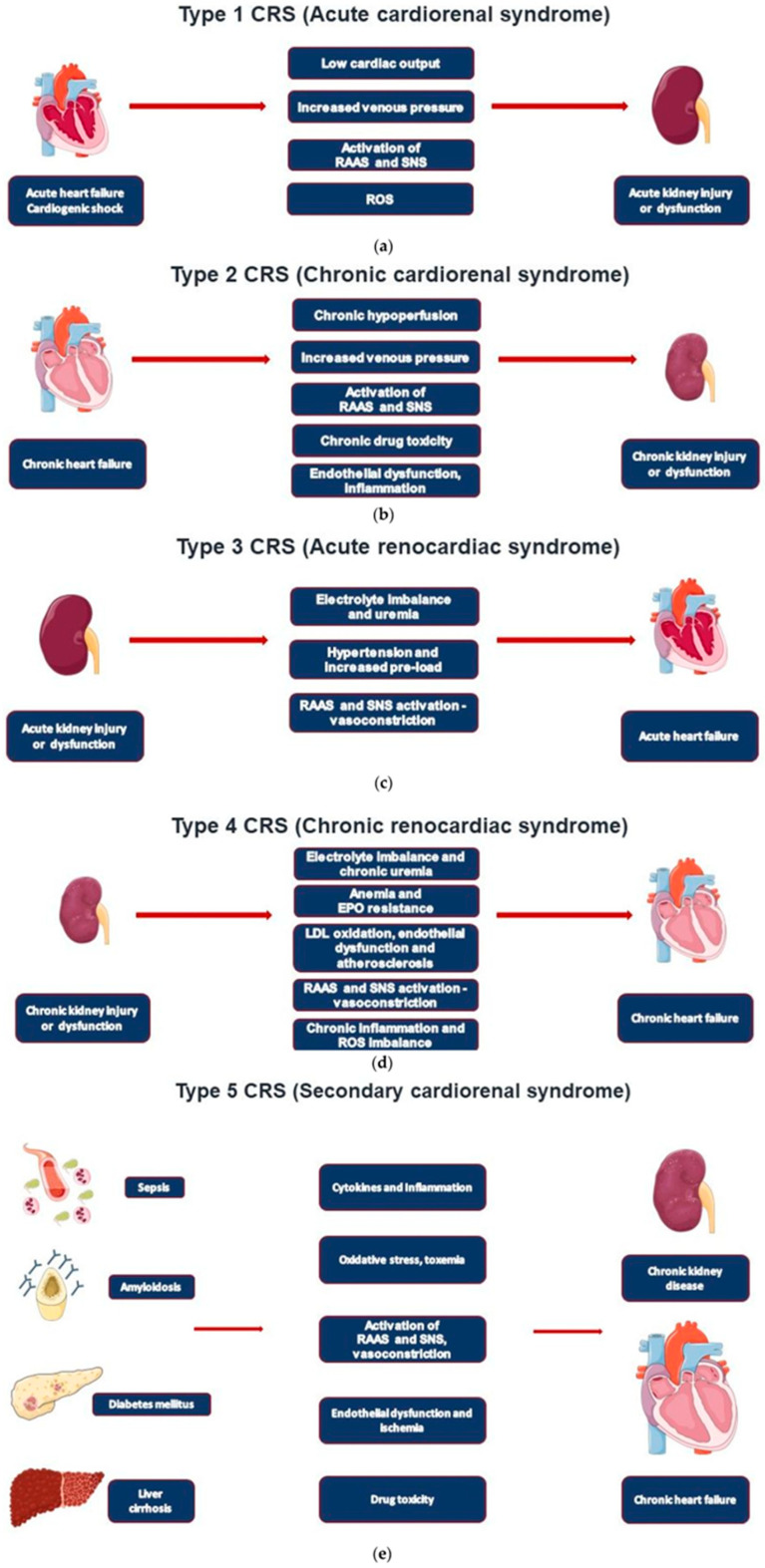
Pathophysiological processes involved in the five cardiorenal syndrome types. Figure obtained from [1].

There are many different individual pathophysiological processes involved in the development of CRS (Figure 1) [1]. Each process must be viewed as one small portion of a larger, more complex pathophysiological mechanism. Given advancements in medicine are increasing life expectancies for the global population, elderly patients will now have a longer cumulative exposure to risk factors of CRS such as diabetes, obesity, hypertension, and other vascular disorders. This suggests that the prevalence of CKD, HF, and subsequently CRS are likely to continue to rise [2,3].

### 1.2. Haemodynamic Alterations and Endothelial Dysfunction

A decrease in cardiac output, in the context of acute HF, can lead to prerenal hypoperfusion. Inadequate blood flow activates the renin–angiotensin–aldosterone system (RAAS) and the sympathetic nervous system (SNS), which promotes fluid retention, increases preload, and induces deterioration of renal function [4]. Elevated venous pressures are increasingly recognised as being an important mechanism in the development of CRS [4]. An adequate pressure gradient is required across the capillary network to maintain blood flow through the vascular system. In acute decompensated HF, an elevation of this pressure reduces the gradient of forward blood flow across the renal vasculature. This slow flow and congestion results in glomerular dysfunction and a decrease in urine output [1,5]. When the body is in a state of fluid overload, excess fluid volume stretches the endothelial lining of vessels, altering their function and inducing stress. This can predispose the body to a harmful cycle which involves renal hypoxia, oxidative stress and inflammation. Inflammatory molecules such as interleukin (IL)-6 and tumour necrosis factor-alpha (TNF-α) increase, worsening the impairment of both the kidneys and the heart [6].

### 1.3. Neurohormonal Activation

The RAAS plays an essential role in CRS, and it is activated to restore organ perfusion [7]. Impaired baroreceptor reflexes, seen in HF, results in the overactivation of the SNS, subsequently increasing renin release from the juxtamedullary cells of the kidneys [8]. Renin stimulates the production of angiotensin II which has multiple systemic effects on the heart, vasculature, and the kidneys [1,2]. There are numerous roles of angiotensin II in the kidneys, but it primarily acts to promote sodium reabsorption. Firstly, it causes an increase in peritubular oncotic pressure and reduction in hydrostatic pressure across the glomerulus via the constriction of renal efferent arterioles, thus leading to the reabsorption of sodium in the proximal tubules [9]. Angiotensin II also has a direct stimulatory effect on the proximal tubular sodium-bicarbonate co-transporters and apical sodium exchangers [9]. Finally, it promotes the reabsorption of sodium in the distal tubules via the activation of aldosterone. Aldosterone increases the expression of endothelin-1 in the kidney, which itself is a potent vasoconstrictor, pro-inflammatory and pro-fibrotic peptide. In chronic activation states, this results in pathological changes and subsequent kidney injury [10]. Adenosine is released in response to an increase in sodium concentration in the distal tubule and results in the reduction in renal blood flow and glomerular filtration rate by constriction of the afferent arterioles. Renin release is promoted by the activation of adenosine type 2 receptors, which enhances sodium reabsorption in the proximal tubule, subsequently reducing diuresis [11]. In response to changes in serum osmolality, arginine vasopressin is synthesised in the hypothalamus and released via the posterior lobe of the pituitary gland. This has effects on the glomerular haemodynamics, arterial blood pressure, and non-haemodynamic renal mechanisms [2]. It has a direct effect via the vasopressin V2 receptors in the collecting duct, leading to water retention and, chronically, the progression of CKD. It also has a direct and indirect contributory effect on the release of renin [12].

### 1.4. Inflammation and Oxidative Stress

In the mitochondria, reactive oxygen species (ROS) are formed as a by-product of cellular metabolism. Once the formation of ROS surpasses the body’s ability to process these by-products, it is termed oxidative stress. The accumulation of ROS results in cellular damage, endothelial dysfunction, and the progression of atherosclerosis [2]. Ischaemic injury, venous congestion, and inflammation, which all occur as CRS establishes itself, trigger further oxidative stress [13]. In HF, the production of adenosine triphosphate (ATP) is mechanistically shifted from fatty acid oxidation to glycolysis, resulting in a decrease in myocardial ATP. This process is insufficient to meet the needs of the heart, and so the threshold for hypoxaemia, apoptosis, and subsequent cell death is lowered [2]. In patients with advanced CKD and end-stage kidney disease (ESKD), there is an abundance of cardiovascular risk factors, due to the contributory effect of uraemic toxins and dialysate solutions, which themselves lead to oxidative stress and subsequent carotid artery intimal thickening and left ventricular hypertrophy [14]. Chronic inflammatory states, such as CKD and HF, can lead to cell death and fibrosis through the generation of pro-inflammatory biomarkers. The activation of the SNS and RAAS axis contributes to the inflammatory cascade and amplified release of pro-inflammatory cytokines TNF-α, TWEAK, IL-1, and IL-6 [2]. C-reactive protein (CRP) itself is an acute phase reactant which can contribute to the process of atherosclerosis [7].

## 2. Diagnostic Challenges of Cardiorenal Syndrome in the Elderly

### 2.1. Signs and Symptoms

CRS poses several diagnostic challenges in the elderly. Primarily, these difficulties are due to the complex nature of the condition and the common comorbidities associated with age. Dyspnoea, fatigue, and signs of fluid overload are symptoms commonly associated with HF [15]. CKD is often incidentally diagnosed through routine investigations but if advanced, patients may report non-specific symptoms such as fatigue, poor appetite, nausea, vomiting, peripheral oedema and dyspnoea [16]. Frailty and various other conditions can present with similar symptoms, and may even mask the symptoms of CRS, making the diagnosis of CRS much more challenging. Elderly patients may present with non-specific symptoms such as delirium, falls or worsening frailty, rather than the organ-specific signs and symptoms one may expect in HF or CKD.

### 2.2. Serum Creatinine

Despite its utility in the diagnosis of acute kidney injury (AKI), serum creatinine in the context of HF can often be challenging to accurately interpret. In a hypervolemic state, serum creatinine levels can be falsely normal or even low. Following diuresis, the concentration of creatinine increases, and this effect can often be mistaken for an AKI resulting in a dose reduction or even a cessation of diuretic treatment [2]. This issue is expanded upon in the therapeutic challenges section later in the review. Serum creatinine can also be affected by reduced muscle mass, protein intake, and elevated levels of inflammation. All of these are more common with chronic health conditions and increased frailty, further muddying the diagnostic waters in CRS [2].

### 2.3. Other Biomarkers in CRS

In addition to serum creatinine, alternative biomarkers have become more widely available for clinical use in recent years. Cystatin C is a low molecular weight protein which is freely filtered at the glomerulus and metabolised in the proximal tubule [17]. It can be used to calculate estimated glomerular filtration rate (eGFR) like creatinine, but is less affected by muscle mass. Cystatin C has its limitations though, as it can be affected by conditions such as thyroid disease, steroid use, smoking and inflammation [18].

The use of urine samples to quantify proteinuria and albuminuria have historically been used to evaluate CKD and its progression. In the context of CRS, where there is no gold standard diagnostic test, this could prove useful to ensure important diagnoses such as amyloidosis or Fabry disease are not missed, which can present with CKD or HF [19].

B-type or Brain Natriuretic Peptide (BNP) can be used clinically to aid in the diagnosis of AKI in CRS patients; however, its sensitivity in the early detection of pathology is limited [20]. As blood pressure or volume overload increases, BNP is synthesised by left ventricular cardiomyocytes. It works by decreasing kidney tubule resistance and increasing the glomerular filtration rate. BNP dampens the function of the SNS, resulting in a reduction in cardiac afterload by relaxing vascular smooth muscle and decreasing blood pressure [21]. Similarly, BNP works against the RAAS axis, reducing renin secretion, which ultimately promotes renal arterial dilation, increases kidney blood flow, and induces a diuretic effect [21]. Chronic activation of the RAAS axis and the SNS can inhibit BNP activity, worsening CRS [20]. In the context of acute HF, patients with more elevated levels of BNP have been found to have worsened hospital outcomes [21]. However, as age and the prevalence of age-related health conditions increase, the levels of BNP also rise, limiting its use as a biomarker in the diagnosis of CRS [20].

### 2.4. Comorbidities and Polypharmacy

As the prevalence of CRS continues to rise across the global population, there needs to be an understanding of comorbidities and medications which can affect the development of this syndrome. Cardiovascular, kidney, and metabolic diseases overlap, and their coexistence has been demonstrated in many epidemiological studies [22]. Type two diabetes mellitus (T2DM) is a significant risk factor in both acute and chronic HF [23], and it is well known to be a leading cause of CKD and ESKD.

Hypertension is known to contribute to both cardiac and renal dysfunction. Hypertension itself can directly damage vessels within the renal and cardiovascular systems, impairing both cardiac output and kidney filtration. It directly affects the heart by increasing the afterload, contributing to left ventricular hypertrophy, which can progress to diastolic or systolic dysfunction. The RAAS and SNS are activated by high blood pressure, resulting in vasoconstriction, sodium retention and further damage to both the kidneys and the heart.

Anaemia of chronic disease is commonly observed in patients with CKD and HF. In CKD, it is associated with poorer outcomes, including cognitive impairment, reduced quality of life, progression of kidney disease, and an increase in mortality [24]. Anaemia itself results in a reduced oxygen supply directly resulting in cell death from ischaemic insults [2].

Polypharmacy can pose as a challenge in the diagnosis and treatment of CRS. It is defined as the use of five or more concurrent drugs and is more common in patients with multiple comorbidities. It is worth noting that reasonable, evidence-based treatments for comorbid conditions seen in CRS, such as diabetes and hypertension, may influence the development of AKI and subsequently make the diagnosis and management of CRS more challenging. For example, patients with HF with reduced ejection fraction should be assessed on an individual basis to potentially commence on the four pillars of HF treatment (RAAS inhibition, beta blockade, mineralocorticoid receptor antagonism, and sodium-glucose co-transporter inhibition), alongside diuretics and anaemia control. The RAAS inhibition aspect (utilising one of angiotensin converting enzyme inhibitors, angiotensin II receptor blockers or angiotensin receptor-neprilysin inhibitors, detailed further in the therapeutic challenges section) may potentially have adverse effects on kidney function [25]. Subsequently, this can contribute to the challenge of diagnosing CRS.

## 3. Therapeutic Challenges of Cardiorenal Syndrome in the Elderly

### 3.1. Diuretics

As noted above, fluid retention and congestion can certainly occur during CRS. Diuretic therapies are a longstanding cornerstone of heart failure (HF) management, recommended in both American [26] and United Kingdom [27] guidelines. In particular, loop diuretics, which function by inhibiting the Na-K-2Cl co-transporter in the thick ascending limb of the Loop of Henle, are commonly used to aid natriuresis and volume loss. Increasingly in modern practice, sequential blockade of the renal tubule with different drug classes is utilised [28], for example, using thiazide or thiazide-like diuretics such as hydrochlorothiazide or metolazone. Although acute creatinine increases are observed in diuretic treatment [29], they are often transient, do not worsen outcomes, and do not mandate cessation of therapy or any dose reduction [30]. In CRS, diuretics improve ventricular filling and enhance renal perfusion [31], hence form part of typical treatment algorithms. Under-diuresis due to fears of worsening kidney function in the context of HF, in particular acute HF, is associated with poor clinical outcomes [31]. Hence, perhaps it would be pertinent to persist with adequate diuresis in acute decompensated HF, even when AKI is present [32].

Unfortunately, diuretic resistance is prevalent in CRS and is associated with a poorer prognosis [32]. Diuretic resistance occurs more frequently in those with more comorbidities, and therefore is particularly relevant to the elderly, with several different mechanisms implicated [33].

Diuretics are associated with several common complications and adverse effects. One of the most well-known is electrolyte imbalances. Loop diuretics can induce hyponatraemia, although at a lower rate compared with thiazide and thiazide-like diuretics, due to their inherent natriuretic properties that clinicians are taking advantage of. However, the finding of hyponatraemia should elicit a fluid assessment and clinical correlation, as it is a common finding in HF itself due to hypervolaemia [34]. Especially in older patients, hyponatraemia may contribute to confusion and delirium [35].

Potentially life-threatening hypokalaemia is another notable risk with many classes of diuretics. Firstly, due to its natriuretic effect, sodium delivery to the distal tubules is naturally increased, intensifying sodium-potassium exchange. Secondly, volume reduction due to the diuretic effect induces secondary hyperaldosteronism, promoting urinary potassium loss [36]. Aside from the cardiac/arrhythmogenic effects of low potassium, hypokalaemia can cause muscle weakness, thereby potentially impairing mobility, subsequently contributing to falls and other consequences of impaired mobility [37]. Electrolyte abnormalities can be exacerbated by several other common medications acting on the RAAS; as noted above, polypharmacy is an important consideration in the elderly.

Thiazide diuretics have been linked with an increased risk of developing T2DM [38], a disease in which its severity is associated with increased comorbid status, with its microvascular (e.g., retinopathy, neuropathy) and macrovascular (e.g., stroke, peripheral arterial disease) sequelae [39]. It is thought that hyperglycaemia itself can predispose the release of ROS, promoting oxidative stress, microvascular and macrovascular disease, and ultimately end-organ damage [22]. These diabetic complications come with well-known risks including cognitive decline and falls, the implications of which are discussed below.

Via a multitude of proposed mechanisms, including inducing orthostatic hypotension and dizziness, diuretic therapy has been implicated as an independent risk factor for falls [40]. It is widely known that falls, especially amongst the elderly population, have a serious negative impact on independence and quality of life. Indeed, falls are the second leading cause of unintentional injury deaths across the globe [41]. Falls can of course result in physical injury, but it is also important to note the impact of extra medications (such as analgesia) and their adverse effects, impact on reducing confidence (and subsequently reduced muscle strength and mobility), and increased risk of hospital admissions (including nosocomial infections) [42].

### 3.2. RAAS Inhibition

As noted earlier in this review, the inability of the heart to generate sufficient prerenal perfusion is intrinsic to certain forms of CRS. As a result, reduced renal afferent flow activates the RAAS axis, leading to fluid retention and worsening pump failure [4]. Because the RAAS plays such a key role in the pathogenesis of CRS, inhibiting the system is a target for treatment modalities.

Cardiorenal protection via RAAS blockade can be obtained with several different classes of drug, such as angiotensin-converting enzyme inhibitors (ACEis), angiotensin receptor blockers (ARBs), mineralocorticoid receptor antagonists (MRAs), and angiotensin receptor-neprilysin inhibitors (ARNIs) [43]. ACEis inhibit the conversion of angiotensin I to II, thereby diminishing its vasoconstriction effects. Similarly, ARBs block the receptor to which angiotensin II binds. MRAs competitively bind to mineralocorticoid receptors, blocking the action of aldosterone, resulting in sodium excretion. Finally, ARNIs utilise both an ARB and a neprilysin inhibitor in combination, the latter of which inhibits the breakdown of BNP, whose favourable effects are detailed earlier in this review. Blockade of the RAAS axis can have favourable outcomes on end-organ damage and subsequent quality of life [44].

However, whilst generally well-tolerated, there are many well-known adverse effects of RAAS inhibition. The older population are at an increased risk of AKI for a variety of reasons, including dehydration, renal parenchymal diseases and drug-related effects [45]. This can be exacerbated by RAAS inhibition.

Hyperkalaemia is a widely known effect of RAAS blockade via inhibition of the effects on aldosterone. In the elderly, plasma renin activity declines, as do plasma aldosterone levels [46]. As a result of this combination of factors, hyperkalaemia risk is significantly increased in an older patient. Increased use of non-steroidal anti-inflammatory drugs (NSAIDs) in this population further contributes to hyperkalaemia risk [45].

Hypotension risk in the elderly population, and its sequelae, is also widely reported with RAAS blockade. The risk of hemodynamic instability with RAAS blockade is further accentuated by the presence of CKD [47]. The subsequent risk of falls has been described above.

Finally, anaemia—of any cause, as well as due to CKD/CRS—is common, and increasingly so as age increases. This contributes significantly to a worse quality of life and has negative effects of morbidity and mortality [48]. There is evidence that the RAAS axis has a key role in regulating erythropoiesis, and inhibiting the axis can potentially exacerbate anaemia and its negative downstream effects.

### 3.3. Sodium-Glucose Co-Transporter 2 Inhibitors

Sodium-glucose co-transporter 2 inhibitors (SGLT2i) are a relatively new drug class, now indicated in management of several conditions. Although initially developed for use in T2DM [49], they have shown significant benefit in HF [50] and CRS. Notably, their morbidity benefit is present regardless of the presence of T2DM, or severity of CKD or HF [51]. There are multiple mechanisms by which SGLT2i function. Fundamentally, they reduce glucose and sodium reabsorption by inhibiting SGLT2 channels in the proximal convoluted tubule (PCT), where around 90% of filtered glucose is reabsorbed [52]. Downstream effects include osmotic diuresis and natriuresis, reduced glucotoxicity of cells, and modulated sodium and calcium ion transport in cardiomyocytes [53,54]. Particularly relevant to CRS is the fact that, because the drugs act on the PCT, they are not typically indicated in patients with eGFR < 20 mL/min/1.73 m^2^, depending on the SGLT2i selected. However, SGLT2i have a role in delaying progression of CRS, irrelevant of type of CRS [55].

One of the more important potential adverse effects of SGLT2i drugs is diabetic ketoacidosis (DKA), and, in particular, atypical DKAs such as euglycaemic DKA. One study noted a ~7-fold increased risk of ketoacidosis with patients using SGLT2i [56]. DKA is a potentially fatal condition, and in the elderly, who have a higher rate of comorbidity and polypharmacy, morbidity and mortality will increase [57].

Another potentially fatal, albeit rare, adverse effect of SGLT2i use is the development of Fournier’s gangrene, which is a urological emergency characterised by necrotising infection of the genitalia [58]. Other risk factors for Fournier’s gangrene include atherosclerosis, peripheral vascular disease, and malnutrition [59], and so although uncommon, it is worth being aware of, especially in an increasingly comorbid population such as the elderly. Of note, it was previously reported that SGLT2i use increased the risk of urinary tract infections and meta-analyses have come to mixed conclusions [60,61,62].

Finally, a further relevant feature of SGLT2i use includes reducing blood pressure—though again, the literature is divided in whether they can induce significant hypotension [63,64], which could lead to falls and other issues.

### 3.4. Renal Replacement Therapy

Finally, separately from medication-based management of CRS, patients may be evaluated for initiation of renal replacement therapy (RRT) in the form of dialysis or ultrafiltration. Benefits of dialysis are relatively clear; the fluid removed contains approximately double the sodium load compared to that in urine produced via diuresis [65,66], as well as control over the rate of fluid removal, enabling accelerated decongestion if tolerated.

There are many factors to consider when choosing between peritoneal dialysis (PD) and haemodialysis (HD), especially in an elderly patient. However, further considerations are needed prior to this, in the form of a decision about whether to initiate dialysis or ultrafiltration in the first place. Indeed, in several studies, earlier initiation of dialysis has been linked with poorer outcomes [67,68]. Secondly, aside from survival impact, there are massive repercussions on quality of life (QoL) for those on dialysis, both acutely [69] and long-term [70], such as increased incidence of fatigue, pain, and sleep disorders, all of which can impact independence, mobility, falls risk, and other aspects. From a clinician’s point of view, an increasing catalogue of comorbidities, such as poor vascular access, malnutrition, or significantly impaired cognitive function, can preclude a patient from being offered RRT [71].

As such, there should be increased efforts to encourage patient-centred care by facilitating well-informed decision-making, as patients may appropriately decline RRT as a result. Properly conducted advance care planning (ACP) is a must.

### 3.5. Exercise Programmes

Exercise programmes for chronic illnesses have been utilised effectively. For example, cardiac rehabilitation is indicated for those who have suffered a myocardial infarction or developed heart failure and has been shown to significantly lower all-cause mortality [72]. Indeed, exercise in general, regardless of whether it is part of a formal programme or not, has been shown to improve QoL and reduce hospitalisation rate [73]. Exercise or regular physical activity is recommended by several professional associations including the European Society of Cardiology [74] and the American Heart Association [26].

### 3.6. Sodium Restriction

Sodium restriction is another recommendation in CRS management guidelines. However, this is somewhat more controversial than exercise. Although key strategies in the management of HF and CRS involve RAAS inhibition, thereby reducing sodium resorption, some studies have shown that attempts to reduce dietary sodium intake can lead to poorer outcomes due to exacerbation of malnutrition [75]. The prevalence of malnutrition is notably higher amongst older adults [76], and so sodium restriction decisions are increasingly relevant in this cohort. Due to these factors, it is clear that a generic treatment plan is not appropriate, and individualised patient care plans are required. Furthermore, these plans should be regularly reviewed, and revisited and altered if necessary.

### 3.7. Fluid Restriction

Fluid restriction is another applied strategy in management for some forms of CRS, although recent evidence has brought the wide application of fluid restriction practices in CRS contexts into question. Common to most treatment guidelines, the general principle of restricting fluid intake is to avoid electrolyte imbalances and reduce congestive symptoms and complications. One meta-analysis indicated a benefit to all-cause mortality and hospitalisation [77], but its methodology has been the subject of critique. Very recently, a randomised-controlled trial found no benefit to fluid restrictions in HF patients and served only to increase thirst distress [78]. Several other smaller reviews and studies have found similar results [79,80], questioning the long-held assertion that fluid restrictions are beneficial in HF and CRS overall. Given that elderly dehydrated patients are six times more likely to die compared to those with a normal hydration status [81], clinicians should be careful not to induce further complications. Once again, fluid restrictions should be considered on an individual case-by-case basis, and may not be suitable for all CRS patients, especially the elderly population.

### 3.8. Optimising Individualised Care Planning and Multi-Disciplinary Team Support

As highlighted throughout the previous sections, decisions around CRS management in the elderly can be complex. Treatment options are limited, and each treatment comes with a variety of possible adverse effects—where each adverse effect comes with its own assortment of sequelae. It is clear that each of these treatments and issues are themselves impacted by an individual’s comorbid state, social circumstances, and polypharmacy. Sometimes, a delicate balance must be struck, and one invaluable tool in finding the sweet spot is utilising different colleagues’ expertise in the form of a multi-disciplinary team (MDT) approach. Input from cardiologists, nephrologists, geriatricians and palliative care doctors may be sought, as well as contributions from other healthcare personnel such as pharmacists, physiotherapists and dietitians. It is apparent that a ‘one-size fits all’ approach to treat CRS, especially in the elderly, is not suitable.

### 3.9. Advance Care Planning

ACP is a shared decision-making process in relation to planning future care and support, that can evolve and change as a patient’s life continues, typically but not exclusively occurring near the end-of-life stages. It is important to discuss personal values and preferences before reaching a point in which a patient is too unwell to make a decision [82]. Ideally involving family and those who are important to the patient, ACP aims to assuage the transition from active treatment to palliation and end-of-life care [83]. More specific to CKD and CRS, ACP may involve decisions related to starting—or withdrawing—RRT, as well as more general principles such as preferred places of care and death. Unfortunately, several studies have noted poor utilisation of ACP by renal clinicians [84,85], citing professional, environmental, and cultural barriers. Furthermore, when ACP discussions are held, they typically might cover decisions related to advanced life support and resuscitation but rarely include decisions around dialysis [86].

With an ageing population, decisions around dialysis withdrawal are likely to become more relevant. Clinicians should be aware of the numerous common barriers to withdrawal and the ‘difficult conversations’ that may ensue, including considering withdrawal as ‘giving up’ or a ‘death sentence’ [87]. It is paramount that ACP conversations—including discussions about dialysis withdrawal—take place while there is enough time for the patient and family to consider their preferences and decisions. The National Institute for Health and Care Excellence Guidance in End-of-Life Care [88] suggests proactive identification of people approaching the end of their life by asking the ‘surprise question’: “Would you be surprised if the patient were to die in next year, months, weeks, days?” More specific to dialysis withdrawal, important considerations include QoL, and inability or unwillingness to tolerate dialysis [89]. Through careful and sensitive dialogue, broaching the subject of dialysis withdrawal can be made much easier.

## 4. Conclusions

**Figure 2:**
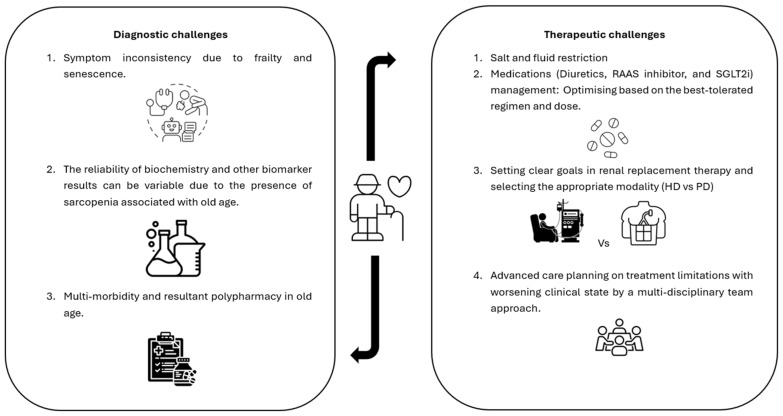
Diagnostic and therapeutic challenges of cardiorenal syndrome in the elderly.

CRS is a complex condition to both diagnose and treat, and this process is made more complicated in those who have a high burden of comorbidities or polypharmacy, particularly the case for many amongst the elderly population (Figure 2). Each therapeutic option comes with its own set of potential adverse effects, and management decisions should be made on a case-by-case basis, utilising MDT support where available. Comprehensive advance care planning for the elderly population with CRS including discussions around RRT withdrawal if indicated, is essential.

## Data Availability

Data sharing is not applicable.

## Abbreviations

The following abbreviations are used in this manuscript:

ACEi	Angiotensin-converting enzyme inhibitor
ACP	Advance care planning
AKI	Acute kidney injury
ARBs	Angiotensin receptor blockers
ARNIs	Angiotensin receptor-neprilysin inhibitors
ATP	Adenosine triphosphate
BNP	Brain natriuretic peptide
CKD	Chronic kidney disease
CRP	C-reactive protein
CRS	Cardiorenal syndrome
DKA	Diabetic ketoacidosis
eGFR	Estimated glomerular filtration rate
ESKD	End-stage kidney disease
HD	Haemodialysis
HF	Heart failure
IL	Interleukin
MDT	Multi-disciplinary team
MRAs	Mineralocorticoid receptor antagonists
NSAIDs	Non-steroidal anti-inflammatory drugs
PCT	Proximal convoluted tubule
PD	Peritoneal dialysis
QoL	Quality of life
RAAS	Renin–angiotensin–aldosterone system
ROS	Reactive oxygen species
RRT	Renal replacement therapy
SGLT2i	Sodium-glucose co-transporter 2 inhibitors
SNS	Sympathetic nervous system
T2DM	Type 2 diabetes mellitus
TNF-α	Tumour necrosis factor-alpha
